# Economic Cycles of Carnot Type

**DOI:** 10.3390/e23101344

**Published:** 2021-10-14

**Authors:** Constantin Udriste, Vladimir Golubyatnikov, Ionel Tevy

**Affiliations:** 1 Department of Mathematics and Informatics, Faculty of Applied Sciences, University Politehnica of Bucharest, Splaiul Independentei 313, Sector 6, 060042 Bucharest, Romania; tevy@mathem.pub.ro; 2 Academy of Romanian Scientists, Ilfov 3, Sector 5, 050044 Bucharest, Romania; 3 Department of Inverse and Ill-Posed Problems, Sobolev Institute of Mathematics SB RAS, Kotpyug Avenue 4, 630090 Novosibirsk, Russia; golubyatn@yandex.ru

**Keywords:** thermodynamic–economic dictionary, Roegenian economics, economic Carnot cycle, ideal income case, economic Van der Waals surface

## Abstract

Originally, the Carnot cycle was a theoretical thermodynamic cycle that provided an upper limit on the efficiency that any classical thermodynamic engine can achieve during the conversion of heat into work, or conversely, the efficiency of a refrigeration system in creating a temperature difference by the application of work to the system. The first aim of this paper is to introduce and study the economic Carnot cycles concerning Roegenian economics, using our thermodynamic–economic dictionary. These cycles are described in both a Q−P diagram and a E−I diagram. An economic Carnot cycle has a maximum efficiency for a reversible economic “engine”. Three problems together with their solutions clarify the meaning of the economic Carnot cycle, in our context. Then we transform the ideal gas theory into the ideal income theory. The second aim is to analyze the economic Van der Waals equation, showing that the diffeomorphic-invariant information about the Van der Waals surface can be obtained by examining a cuspidal potential.

## 1. Thermodynamic-Economic Dictionary

Thermodynamics is important as a model of phenomenological theory, which describes and unifies certain properties of different types of physical systems. There are many systems in biology, economics and computer science, for which a similar and unitary-phenomenological organization would be desirable. Our purpose is to present certain features of Roegenian economics that are inspired by thermodynamics. In this context, we offer again a morphism of dictionary type that reflects a thermodynamics–economics transfer. The formal analytical-mathematical analogy between economics and thermodynamics is accepted by economists and physicists as well (what differs are the dictionaries used). Starting from these remarks, the papers of Udriste and coauthors [1,2,3,4,5] build a morphism of dictionary type between thermodynamics and economics, admitting that fundamental laws in thermodynamics are transferred into laws for economics.

In the following, we reproduce again the correspondence between the characteristic state variables and the laws of thermodynamics with the macro-economics ingredients as described in Udriste et al. (2002–2019) based on the theory on which the economics was founded in 1971 [6]. They also allow us to include in the economics the idea of a “black hole” with a similar meaning to the one in astrophysics [5].

**Remark** **1.** *(i) Our dictionary started from Roegen’s idea [6,7] that the entropy in thermodynamics must correspond to the entropy in economics. Mimkes’ economics–thermodynamics dictionary [8,9] uses the idea that “production function” corresponds to “entropy”. Obviously, the two dictionaries are different.*

*(ii) In this paper it is only a morphism based on an initial dictionary conceived by Udriste’s research team. Different parts of this dictionary (morphism) can be found in the papers [1,5] and references therein. A full discussion of the thermodynamics–economics morphism will be produced in a future paper.*

*(iii) The marginal inclination to investment turnover can measure the marginal process in changing the capital into a new investment type in the system. This change normally gives better performances to systems in terms of growth and producing more wealth.*

       THERMODYNAMICS
       ECONOMICSU = internal energy…  G = growth potentialT = temperature…I = internal politics stabilityS = entropy…E = entropyP = pressure…P = price level (inflation)V = volume…Q = volume, structure, qualityM = total energy (mass)…Y = national income (income)Q = electric charge…I = total investmentJ = angular momentum…J = economical investment angular momentum       (spin)
       (investment spin)M = M(S,Q,J)…Y = Y(E,I,J)$\Omega =\frac{\partial M}{\partial J}$ = angular speed…$\frac{\partial Y}{\partial J}$ = marginal inclination to investment turnover$\Phi =\frac{\partial M}{\partial Q}$ = electric potential…$\frac{\partial Y}{\partial I}$ = marginal inclination to investment$T_{H}=\frac{\partial M}{\partial S}$ =Hawking temperature…$\frac{\partial Y}{\partial E}$ = marginal inclination to entropy$\mu_{k}$ = chemical potentials…$\nu_{k}$ = economical potentials

The Gibbs–Pfaff fundamental equation in thermodynamics

$$dU-TdS+PdV+\sum_{k}\mu_{k}dN_{k}=0$$

is changed to the Gibbs–Pfaff fundamental equation of economics

$$dG-IdE+PdQ+\sum_{k}\nu_{k}dN_{k}=0.$$

These equations are combinations of the first law and the second law (in thermodynamics and economics, respectively). The third law of thermodynamics $\lim_{T\to 0}S=0$ suggests the third law of economics $\lim_{I\to 0}E=0$ “if the internal political stability *I* tends to 0, the system is blocked, meaning entropy becomes E=0, equivalent to maintaining the functionality of the economic system we must cause disruption”.

**Remark** **2.** *The first law of thermodynamics is equivalent to the law of conservation of energy: Energy cannot be created or destroyed; the total amount of energy in the Universe is fixed. Energy can be transformed from one form to another or transferred from one place to another but the total energy must remain unchanged.*

*The thermodynamics–economics morphism is not surjective (the dictionary is not isomorphism). That is why the sentence “in Thermodynamics there is a law of energy conservation, while in Economics there is no such conservation law” is meaningless.*

Process variables W= mechanical works and *Q* = heat are introduced into elementary mechanical thermodynamics by dW=PdV (the first law) and by elementary heat, respectively, dQ=TdS, for reversible processes, or dQ<TdS, for irreversible processes (second law). Their correspondences in economics,

W=wealthofthesystem,q=productionofgoods

are defined by dW=Pdq (elementary wealth in the economy) and dq=IdE or dq<IdE (the second law or the elementary production of commodities). A commodity is an economic good, a product of human labour, with a utility in the sense of life, for sale-purchase on the market in the economy.

Sometimes a thermodynamic system is found in an external electromagnetic field $\left(\vec{E},\vec{H}\right)$. The external electric field $\vec{E}$ determines the polarization $\vec{P}$ and the external magnetic field $\vec{H}$ determines magnetizing $\vec{M}$. Together they give the total elementary mechanical work $dW=P\;dV+\vec{E}\;d\vec{P}+\vec{H}\;d\vec{M}$. Naturally, an economic system is found in an external econo-electromagnetic field $\left(\vec{e},\vec{h}\right)$. The external investment (econo-electric) field $\vec{e}$ determines initial growth condition field (econo-polarization field) $\vec{p}$ and the external growth field (econo-magnetic field) $\vec{h}$ causes growth (econo-magnetization) $\vec{m}$. All these fields produce the elementary mechanical work $dW=P\;dQ+\vec{e}\;d\vec{e}+\vec{h}\;d\vec{m}$. The economic fields introduced here are imposed on the one hand by the type of economic system and on the other hand by the policy makers (government, public companies, private firms, etc.).

The long term association between thermodynamics and economics can be strengthened with new tools based on the previous dictionary. Of course, this idea of a new thermodynamics–economics dictionary (morphism) produces concepts different from those in econophysics [10] and concepts of thermodynamics in economic systems like in [9,11]. Econophysics seems to build similar economic notions to physics, as if those in the economy were not enough.

The thermodynamics–economics dictionary (morphism) allows the transfer of information from one discipline to its image (Udriste et al. [1,5]), keeping the background of the first discipline that we think was suggested by Roegen in 1971 [6].

**Definition** **1.** *Economics based on rules similar to those in thermodynamics is called Roegenian economics.*

Economics described by previous Gibbs–Pfaff equation is Roegenian economics.

A macro-economic system based on a Gibbs–Pfaff equation is both Roegenian and controllable (see [12]).

Roegenian economics (also called bioeconomics by Georgescu–Roegen) is both a transdisciplinary and an interdisciplinary field of academic research addressing the interdependence and coevolution of human economies and natural ecosystems, both intertemporally and spatially. The economic entropy is closely related to disorder and complexity [7].

## 2. What Is the Carnot Cycle in Thermodynamics?

The Carnot cycle [13,14,15] is a theoretical thermodynamic cycle proposed by French physicist Sadi Carnot. It provides an upper limit on the efficiency that any classical thermodynamic engine can achieve during the conversion of heat into work, or conversely, the efficiency of a refrigeration system in creating a temperature difference by the application of work to the system.

A heat engine is a device that produces motion from heat and includes gasoline engines and steam engines. These devices vary in efficiency. The Carnot cycle describes the most efficient possible heat engine, involving two isothermal processes and two adiabatic processes. It is the most efficient heat engine that is possible within the laws of physics.

The second law of thermodynamics states that it is impossible to extract heat from a hot reservoir and use it all to do work; some must be exhausted in a cold reservoir. Or, in other words, no process can be “one hundred percen” efficient because energy is always lost somewhere. The Carnot cycle sets the upper limit for what is possible, for what the maximally efficient engine would look like.

When we go through the details of the Carnot cycle, we should define two important adjectives, isothermic and adiabatic, which characterize a process. An isothermic process means that the temperature remains constant and the volume and pressure vary relative to each other. An adiabatic process means that no heat enters or leaves the system to or from a reservoir and the temperature, pressure, and volume are all free to change, relative to each other.

**Remark** **3.** *The idea of Carnot’s cycles in the Roegenian economy was suggested by Vladimir Golubyatnikov in a discussion held at the “The XII-th International Conference of Differential Geometry and Dynamical Systems (DGDS-2018), 30 August–2 September 2018, Mangalia, Romania” on the communication of Constantin Udriste about the Roegenian economy and the dictionary that creates this point of view in the economy. The first ideas in this direction were included in a manuscript posted in the arXiv [16].*

## 3. Economic Carnot Cycle, Q−P Diagram

Let us consider again the Roegenian economics (an economic distribution) generated by the Gibbs–Pfaff fundamental equation dG−IdE+PdQ=0 on $R^{5}$, where (G,I,E,P,Q) are economic variables, according the previous dictionary (*G* = growth potential, *I* = internal politics stability, *E* = entropy, *P* = price level (inflation), *Q* = volume, structure, quality).

The heat *Q* in thermodynamics is associated to q=productionofgoods in economics. Let *I* be the internal politics stability, *P* be the price level, and *E* the economic entropy.

**Figure 1:**
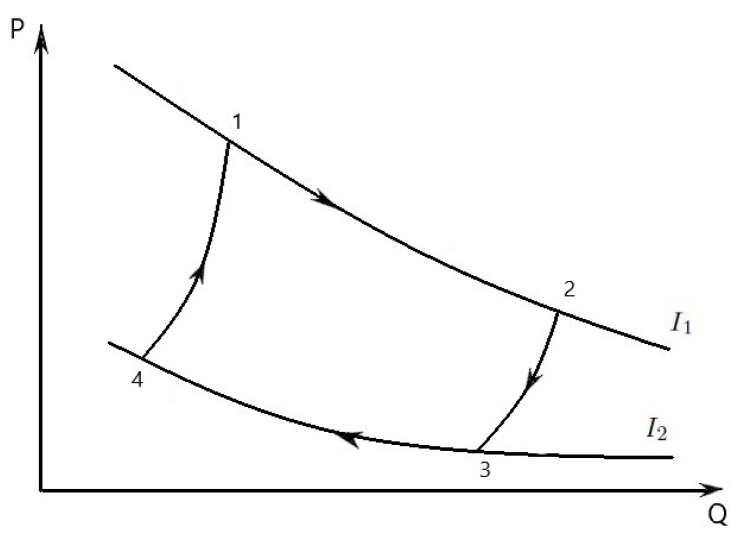
Economic Carnot cycle illustrated on a Q-P diagram to show the wealth done.

The properties of an economic cycle are represented firstly on the Q−P diagram (Figure 1, arrows clockwise). This diagram is based on economical quantities *Q* and *P* that we can measure. In the Q−P diagram, the curves (evolutions) 1-2, 3-4 are considered at constant internal politics stability (corresponding to isothermic transformations) and the curves (evolutions) 2-3, 4-1 are considered at constant levels of goods production regarding as supply (corresponding to adiabatic transformations). The convexity to the right of these curves is opposite to similar curves in thermodynamics (convexity to the left).

Let us describe the four steps that make up an economic Carnot cycle. Process 1-2 is an iso-internal politics stability expansion, where the volume increases and only price level of some goods decreases at a constant internal politics stability. Process 2-3 is a constant levels of goods production expansion.

**Remark** **4.** *The process is microeconomic and it involves only a certain number of goods. The process becomes macroeconomic when we consider aggregate variables, in other words, Volume is associated to Total Production. In this stage we can denote with P the general level of prices (i.e., inflation) and can explain Economic Carnot Cycle as well.*

Process 3-4 is an iso-internal politics stability compression, where the volume decreases and the price level increases at an internal politics stability. Lastly, process 4-1 is a constant levels of goods production compression. Process 2-3 is a constant levels of goods production expansion. Process 3-4 is an iso-internal politics stability compression, where the volume decreases and the price level increases at an internal politics stability. Lastly, process 4-1 is a constant levels of goods production compression.

The useful wealth *W* in the economy that comes out of the economic Carnot cycle is the difference between the wealth $W_{1}$ in the economy done by the economic system in stages 1-2 and 2-3 and the wealth $W_{2}$ in the economy done (or the economic energy wasted) by you in stages 3-4 and 4-1. The economic Carnot cycle described above is the most favorable case, because it produces the largest difference between these values allowed by the laws of economics.

## 4. Economic Carnot Cycle, E−I Diagram

Let (G,I,E,P,Q) be economic variables, according the previous dictionary (*G* = growth potential, *I* = internal politics stability, *E* = entropy, *P* = price level (inflation), *Q* = volume, structure, quality). We refer again the Roegenian economics (an economic distribution) generated by the Gibbs–Pfaff fundamental equation dG−IdE+PdQ=0 on $R^{5}$.

**Figure 2:**
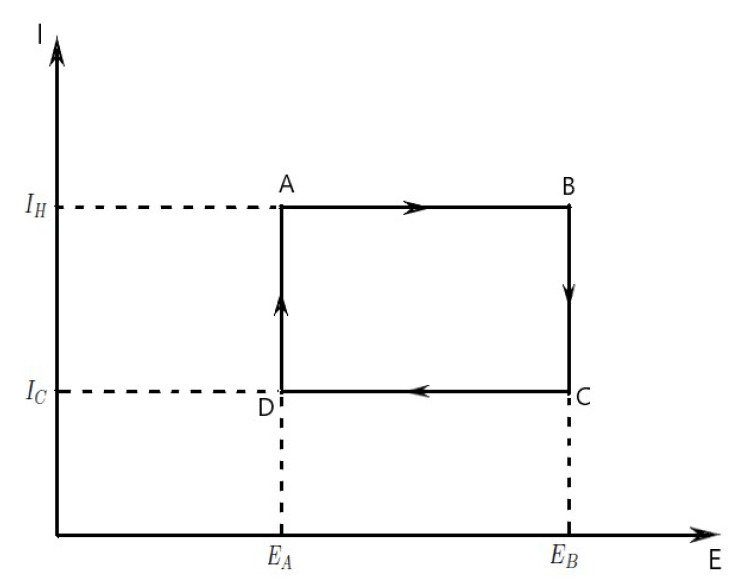
Economic Carnot cycle acting as an economic process with an engine producing goods, illustrated on a E-I diagram.

The heat *Q* in thermodynamics is associated to q=productionofgoods in economics. Let *I* be the internal politics stability, and *E* the economic entropy satisfying the Pfaff equation dq=IdE. The properties of an economic process are represented secondly on the E−I diagram (Figure 2), although *E* and *I* are not directly measurable.

The evolution in the plane E−I of an economic system in time will be described by a curve connecting an initial state (A) and a final state (B). The area under the curve will be

$$q=\int_{A}^{B}I\;dE,$$

which is the amount of economical investments (economical energy) transferred in the process. If the process moves to greater entropy, the area under the curve will be the amount of production of goods absorbed by the system in that process. If the process moves towards lesser entropy, it will be the amount of production of goods removed. For any cyclic process, there will be an upper portion of the cycle and a lower portion. For a clockwise cycle, the area under the upper portion will be the economical energy absorbed during the cycle, while the area under the lower portion will be the economical energy removed during the cycle.

The area inside the cycle will then be the difference between the two, but since the economical energy of the system must have returned to its initial value, this difference must be the amount of the wealth of the economic system over the cycle.

**Theorem** **1.** *The amount of the wealth of the economic system done over a cyclic process is*

$$W=\oint IdE=\left(I_{H}-I_{C}\right)\left(E_{B}-E_{A}\right).$$

**Proof.**  Referring to “*W* = the wealth of the system, *P* = price level (inflation), *Q* = volume, structure, quality”, mathematically, for a reversible process we may write the amount of the wealth of the economic system done over a cyclic process as

W=∮PdQ=∮dq−dG=∮IdE−dG=∮IdE−∮dG=∮IdE.

Since dG is an exact differential, its integral over any closed loop is zero and it follows that the area inside the loop on a E−I diagram is equal to the total amount of the wealth performed if the loop is traversed in a clockwise direction, and is equal to the total amount of the wealth done on the system as the loop is traversed in a counterclockwise direction.

Evaluation of the above integral is particularly simple for the Carnot cycle if we use the E−I diagram (Figure 2). The amount of economic energy transferred as wealth of the system is (see Green formula)

$$W=\oint PdQ=\oint IdE={\;\int \int \;}_{\Delta }dI\;dE=\left(I_{H}-I_{C}\right)\left(E_{B}-E_{A}\right).$$

□

The total amount of economic energy transferred from the production to the system is given by $q_{H}=I_{H}\left(E_{B}-E_{A}\right)$, and the total amount of economic energy transferred from the system to the consumption is

$$q_{C}=I_{C}\left(E_{B}-E_{A}\right),$$

where $E_{B}$ is the maximum system entropy, $E_{A}$ is the minimum system entropy.

## 5. Efficiency of an Economic System

We recall the notations: W is the wealth done by the system (economic energy), $q_{C}$ is the production of goods taken from the system (economic energy leaving the system), $q_{H}$ is the production of goods put into the system (economic energy entering the system), $I_{C}$ is the absolute internal politics stability of the consumption, $I_{H}$ is the absolute internal politics stability of the production.

**Definition** **2.** *The number*

$$\eta =\frac{W}{q_{H}}=1-\frac{I_{C}}{I_{H}}$$

*is called the efficiency of an economic system.*

The absolute internal politics stability of the consumption can not be as small as it is limited by social restrictions. The absolute internal politics stability of the production can not be as large as it is limited by resources and labour. A wrong interpretation of the efficiency of an economic Roegenian system can generate Homeland Falcons Hymn: To grow strong and big without eating anything.

This definition of efficiency makes sense for an economic “engine”, since it is the fraction of the economic energy extracted from the production and converted to wealth done by the system (economic energy). In practical aspects of this theory, we use approximations.

Economic Carnot cycle has maximum efficiency for reversible economic “engine”.

The economic Carnot cycle previously described is a totally reversible cycle. That is, all the processes that comprise it can be reversed, in which case it becomes the consumption Carnot cycle. This time, the cycle remains exactly the same except that the directions of any production and wealth interactions are reversed. The Q−P diagram of the reversed economic Carnot cycle is the same as for the initial economic Carnot cycle except that the directions of the processes are reversed.

## 6. Economic Carnot Cycle—Problems and Solutions

A comparison between the units of measurement in thermodynamics and economics is made in the paper [17].

**1**. If financial assets market (production of goods) absorbed by the engine is $q_{1}$ = 10,000 c.u. (conventional units), what is the wealthy of the system done by the economic Carnot engine?

**Known**: The absolute internal politics stability of the consumption $I_{C}=400\%$, the absolute internal politics stability of the production $I_{H}=800\%$, the financial assets market (production of goods) input $q_{1}$ = 10,000 c.u.

**Wanted**. Wealthy of the system done by economic Carnot engine *W*.

**Solution**. The efficiency of the economic Carnot engine $e=\frac{I_{H}-I_{C}}{I_{H}}$, $e=\frac{1}{2}$.

Wealthy of the system done by economic Carnot engine: $W=eq_{1}$, *W* = 5000 c.u.

**2** The absolute internal politics stability of the production is $I_{H}=600\%$ and the absolute internal politics stability of the consumption is $I_{C}=400\%$. If the wealthy done by engine is *W*, what is the financial assets market output?

**Known**: The absolute internal politics stability of the consumption $I_{C}=400\%$, the absolute internal politics stability of the production $I_{H}=600\%$.

**Wanted**. Financial assets market output $q_{2}$.

**Solution**. The efficiency of economic Carnot engine is $e=\frac{I_{H}-I_{C}}{I_{H}}$, $e=\frac{1}{3}$. The wealthy of the system done by economic Carnot engine is $W=eq_{1}$, $3W=q_{1}$. It follows the stock market: $q_{2}=q_{1}-W=2W$.

**3** An economic Carnot engine has an efficiency of 0.3. Its efficiency is to be increased to 0.5. By what must the internal politics stability of the source be increased if the sink is at 300%?

**Solution** Efficiency is given by $e=1-\frac{I_{2}}{I_{1}}$. Here e=0.3. From $0.3=1-\frac{300}{I_{1}}$, it follows $I_{1}=428.6\%$. For increased efficiency of 0.5, the source internal politics stability should be $0.5=1-\frac{300}{I_{1}}$, i.e., $I_{1}=600\%$. Hence the source internal politics stability should be increased by 600−428.6=171.4 (%).

## 7. Ideal Income Case

To transform the ideal gas theory into the ideal income theory, we recall a part of dictionary (morphism) and we complete it with new correspondences: *Q* = heat ↔ *q* production of goods; *T* = temperature ↔ *I* = internal politics stability; *P* = pressure ↔ *P* = price level; *S* = entropy ↔ *E* = economic entropy; *V* = volume ↔ *Q* = volume, structure, quality; *U* = internal energy ↔ *G* = growth potential; mole ↔ economic mole; *R* = molar gas constant ↔ *R* = molar income constant; *f* = number of degrees of freedom ↔ *f* = number of degrees of freedom. Moreover, we look at Q−P diagram.

Suppose the three states of an economy “inflation, monetary policy as liquidity, income” are reduced to one and the same “income”. By the equipartition theorem, the growth potential of one mole of an ideal income is $G=\frac{f}{2}\;R\;I$, where *f*, the number of degrees of freedom, is 3 for a mono-atomic income, 5 for a diatomic income, and 6 for an income of arbitrarily shaped economic molecules. The quantity *R* is the molar income constant. For the present discussion it is important to notice that *G* is a function of *I* only.

In Figure 1 the economic Carnot cycle is represented in a Q−P diagram. Consider the compression (decreasing “volume, structure, quality”) of the income along the path 3-4. Along this path the internal politics stability is $I_{c}$. The economic work done is converted into production of goods $q_{c}$. This process is so slow that the internal politics stability remains constant (i.e., it is an iso-ips), and hence the the potential growth *Q* of the income does not change, i.e., dQ=0. By the first and second law of economics, reference to Figure 1, and the ideal income law PQ=RI (for the sake of argument we consider one mole of income), we have

$$q_{c}=I_{c}\int_{3}^{4}dE=\int_{3}^{4}PdQ=RI_{c}\int_{3}^{4}\frac{dQ}{Q}\;\;\Rightarrow \;\;E_{4}-E_{3}=R\ln\;\frac{Q_{4}}{Q_{3}}<0.$$

The economic entropy *E* of an ideal income is a logarithmic function of its “volume, structure, quality” *Q*.

In the Q−P plane, the iso-ips 3-4 has the following expression $P=\frac{R\;I_{c}}{Q}$, i.e., *P* is a function of *Q* only.

At point 4, we move on a curve similar to an adiabatic curve. We move inward from $Q_{4}$ to $Q_{3}$ and by this compression the income is amplified to exactly the internal politics stability $I_{h}$. From 4 to 1 the path in the Q−P diagram is similar to adiabatic and reversible path. The entropy does not change during this compression, i.e., dE=0 (an isentropic economic process). It is easy to derive the equation for the adiabatic in the Q−P plane,

$$dG=\frac{f}{2}R\;\;dI=-P\;\;dQ=-\frac{RI}{Q}\;dQ\;\;\Rightarrow \;\;\frac{f}{2}\ln\frac{I_{3}}{I_{4}}=-\ln\frac{Q_{3}}{Q_{4}}$$

and the ideal income law PQ=RI (for the sake of argument we consider one mole of income) implies

$$\frac{f}{2}\ln\frac{P_{3}Q_{3}}{P_{4}Q_{4}}=-\ln\frac{Q_{3}}{Q_{4}}\;\;\Rightarrow \;\;\frac{P_{3}}{P_{4}}={\left(\frac{Q_{3}}{Q_{4}}\right)}^{-\frac{f+2}{f}}.$$

For a mono-atomic income (f=3) the expression of the curve 4-1 is $P=c\;\;Q^{-\frac{5}{3}}$, where $c=P_{4}Q_{4}^{\frac{5}{3}}$.

The integral $\int_{3}^{1}P\;dQ$ is the economic work done. In the Q−P diagram this integral is the negative of the area bounded by the curve 3-4-1 and the *Q* axis.

At point 1, we have the internal politics stability $I_{h}$ and total production of goods $q_{h}$. The iso-ips 1-2 has the expression $P\left(Q\right)=\frac{R\;I_{h}}{Q}$ and the entropy increases, i.e.,

$$E_{2}-E_{1}=R\ln\frac{Q_{2}}{Q_{1}}>0.$$

From 2 to 3 isentropic economic expansion occurs, the internal politics stability decreases to $I_{c}$. The integral $\int_{1}^{3}P\;dQ$ is the economic work. In the Q−P diagram this integral is the area bounded by the curve 1-2-3 and the *Q* axis. Note that this area is larger than the area below the curve 3-4-1. Difference of the two areas, the economic work done during the cycle, is ∮PdQ, where the closed curve is 1-2-3-4-1.

By the first law of economics, the total work *W* is equal to $q_{h}-q_{c}$.

The economic process, as depicted in Figure 1, runs clockwise. All the economic processes are reversible, so that all arrows can be reverted.

## 8. Economic Van der Waals Equation

Let $Q_{m}$ be the molar volume of the income, *R* be the universal income constant, *P* be the price level, *Q* be the “volume, structure, quality”, and *I* be internal politics stability.

The Van der Waals equation in Thermodynamics [18] has a correspondent in economics, where the variables are P,Q,I. The idea is based on plausible reasons that real incomes do not follow the ideal income law.

The ideal income law states that volume *Q* occupied by *n* moles of any income has a price level *P*, typically in reference currency, at internal politics stability *I* in percents—see, for instance, Moody’s rating. The relationship for these variables, PQ=nRI, is called the ideal income economic law or equation of state.

The economic Van der Waals equation is

$$\left(P+\frac{a}{Q_{m}^{2}}\right)\left(Q_{m}-b\right)=R\;I,\;\;\mathrm{or}\;\;\left(P+\frac{an^{2}}{Q^{2}}\right)\left(Q-nb\right)=n\;R\;I.$$

when the molar volume $Q_{m}$ is large, the constant *b* becomes negligible in comparison with $Q_{m}$, the term $\frac{a}{Q_{m}^{2}}$ becomes negligible with respect to *P*, the economic Van der Waals equation reduces to the ideal income economic law, $P\;Q_{m}=R\;I$.

The economic Van der Waals equation successfully approximates the behavior of real “mp−lc = monetary policies as liquidity or consumption” above critical internal politics stability and is qualitatively reasonable for points (mp−lc, level of prices, internal politics stability) around critical point. However, near the transitions between income and mp−lc, in the range of P,Q,I, where the mp−lc phase and the income phase are in equilibrium, the Van der Waals equation fails to accurately model observed experimental behaviour; in particular *P* is a constant function of *Q* at given internal politics stability, but the Van der Waals equation do not confirm that. As such, the Van der Waals model is not useful only for calculations intended to predict real behavior in regions near the critical point, but also for qualitatively properties. Empirical corrections to address these predictive deficiencies can be inserted into the Van der Waals model, e.g., equal area rule (see Maxwell theory), and related but distinct theoretical models, e.g., based on the principle of corresponding states. All of these permit to achieve better fits to real mp−lc behaviour in equations of more comparable complexity.

**Figure 3:**
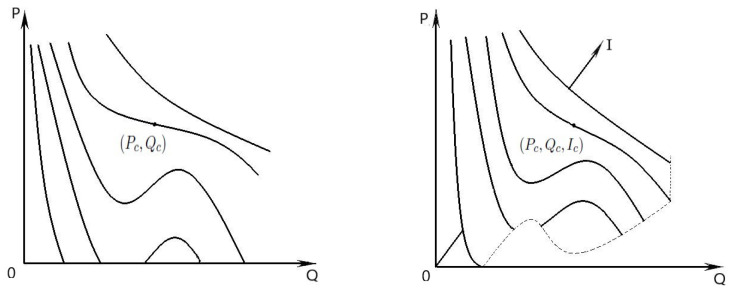
Van der Waals surface.

Geometrically, the previous equation represents a surface located in the first octane of the point space ${\left(0,\infty \right)}^{3}=\left\{\left(P,Q,I\right)\right\}$, that we call the economic Van der Waals surface, Figure 3. The shape of this surface follows from the shapes of the sections by the planes *I* = constant (Van der Waals iso-“mp-lc”). There is a stationary inflection point in the constant—“internal politics stability” curve (critical iso-“mp-lc”)—on a Q−P diagram. This means that at the degenerate critical point we must have

$$\frac{\partial P}{\partial Q}{|}_{I}=0,\;\;\frac{\partial^{2}P}{\partial Q^{2}}{|}_{I}=0.$$

On the Van der Waals surface, the above critical point $\left(P_{c},Q_{c},I_{c}\right)$ has the coordinates

$$P_{c}=\frac{a}{27b^{2}},\;\;Q_{c}=3b,\;\;I_{c}=\frac{8a}{27bR}.$$

**Remark** **5.** *Let us write the economic Van der Waals surface in the implicit form W:f(P,Q,I)=0. Eliminating the critical point, the Gaussian curvature of this surface can be written as the determinant*

$$K=-\frac{1}{{∥\nabla f∥}^{4}}\left|\begin{array}{cccc} f_{PP} & f_{PQ} & f_{PI} & f_{P}\\ f_{QP} & f_{QQ} & f_{QI} & f_{Q}\\ f_{IP} & f_{IQ} & f_{II} & f_{I}\\ f_{P} & f_{Q} & f_{I} & 0 \end{array}\right|,$$

*where the inferior indices mean partial derivatives.*

**Remark** **6.** *Let us show that we can find the critical point without the use of the “partial derivative”. At the critical point, the income is characterized by the critical values of $I_{c}$, $P_{c}$, $Q_{c}$, which are determined only by the income properties. From the algebraic point of view, the iso-“mp-lc” through the critical point, i.e., $Q^{3}-\left(b+\frac{RI}{P}\right)Q^{2}+\frac{a}{P}\;\;Q-\frac{ab}{P}=0,$ like equation in Q, has a triple real root $Q_{c}$, i.e., the equation should be identified to ${\left(Q-Q_{c}\right)}^{3}=0$. Expanding this cube and equating the coefficients of the terms with equal powers of Q, we find the expressions for the critical parameters.*

*This phenomena can be explain in terms of economic collapse of a certain number of variables named technically “Fundamentals”. This event is perfectly modeled by the previous Gaussian curvature and the Hessian meaning.*

Let’s connect with catastrophe theory. For this we consider the function $\varphi :{\left(0,\infty \right)}^{3}\to R^{3},$ of components

$$x=\frac{1}{Q}-\frac{1}{3b},\;\;\alpha =\frac{P}{a}+\frac{RI}{ab}-\frac{1}{3b^{2}},\;\;\beta =-\frac{P}{3ab}+\frac{RI}{3ab^{2}}-\frac{2}{27b^{3}}.$$

One remarks that $\varphi :{\left(0,\infty \right)}^{3}\to \varphi \left({\left(0,\infty \right)}^{3}\right)$ is a global diffeomorphism and $\varphi \left(P_{c},Q_{c},I_{c}\right)=\left(0,0,0\right)$. The image of economic Van der Waals surface by φ is characterized by the equation $x^{3}+\alpha x+\beta =0,$ where

$$x\in \left(-\frac{1}{3b},\infty \right),\;\;\alpha \in \left(-\frac{1}{3b^{2}},\infty \right),\;\;\beta \in \left(-\infty ,\frac{-2}{27b^{3}}\right)\cup \left(\frac{-2}{27b^{3}},\infty \right),$$

that is, it is part of the cuspidal catastrophe manifold.

**Theorem** **2.** *All diffeomorphic-invariant information about Van der Waals mp−lc can be obtained by examining the cuspidal potential $x\to f\left(x,\alpha ,\beta \right)=\frac{1}{4}x^{4}+\frac{1}{2}\alpha x^{2}+\beta x,$ which is equivalent to Gibbs’ free energy.*

## 9. Conclusions

Thermodynamics is not Economics, and Economics is not Thermodynamics.

Some general concepts of the thermodynamics are transferred to economics and biology through appropriate dictionaries (morphisms). This means that economics or biology are thought as collections of notions, and the relationships between them are borrowed from thermodynamics. So far, no one has set out to determine notions of thermodynamics coming from economics or biology.

The economic Carnot cycle in Roegenian economics, introduced and analyzed in the Section 3, Section 4, Section 5 and Section 6, is not the same with economic cycles described in the papers [13,19,20,21]. According these papers, a business cycle is a pattern of economic booms and busts exhibited by the modern economy. Sometimes referred to as a trade or economic cycle, a business cycle is the measured expansion and contraction of economic growth within a period. Business cycles naturally fluctuate through four phases or stages: Expansion, peak, contraction and trough.

In our sense, an economic Carnot cycle in Roegenian economics (a totally reversible cycle) is the counterpart of the Carnot cycle in thermodynamics, obtained by a suitable dictionary (morphism). This Carnot-type economic cycle was created through a dictionary-like morphism that we used in our papers. Any other comment is superfluous.

The Q−P and E−I diagrams are useful as visual aids in the analysis of ideal power cycles in Roegenian economics.

It is hoped that the paper will open up new avenues for future work on macro and micro systems.
